# Constraining barrow entropy-based cosmology with power-law inflation

**DOI:** 10.1140/epjc/s10052-023-11499-7

**Published:** 2023-04-26

**Authors:** G. G. Luciano

**Affiliations:** Applied Physics Section of Environmental Science Department, Escola Politècnica Superior, Universitat de Lleida, Av. Jaume II, 69, 25001 Lleida, Spain

## Abstract

We study the inflationary era of the Universe in a modified cosmological scenario based on the gravity-thermodynamics conjecture with Barrow entropy instead of the usual Bekenstein–Hawking one. The former arises from the effort to account for quantum gravitational effects on the horizon surface of black holes and, in a broader sense, of the Universe. First, we extract modified Friedmann equations from the first law of thermodynamics applied to the apparent horizon of a Friedmann–Robertson–Walker Universe. Assuming a power-law behavior for the scalar inflaton field, we then investigate how the inflationary dynamics is affected in Barrow cosmological setup. We find that the inflationary era may phenomenologically consist of the slow-roll phase, while Barrow entropy is incompatible with kinetic inflation. By demanding observational consistency of the scalar spectral index and tensor-to-scalar ratio with recent Planck data, we finally constrain Barrow exponent to $\Delta \lesssim \mathcal {O}(10^{-4})$, which is the most stringent bound in so-far literature.

## Introduction

The effort to understand the statistical mechanics of black holes [1] has opened up new scenarios in modern theoretical physics, including the study of the AdS/CFT correspondence [2, 3] and the investigation of the connection between gravity and thermodynamics. Beyond their intrinsic interest, both these two lines of research might potentially have a deep impact upon the development of quantum gravity, mainly because they are the most successful realizations of the holographic principle [4, 5]. While the AdS/CFT correspondence is based on the description of the background geometry in terms of anti-de Sitter vacuum solutions, the interplay between gravity and thermodynamics finds its conceptualization in the so-called *gravity-thermodynamics* conjecture [6–8], which states that Einstein field equations are nothing but the gravitational counterpart of the laws of thermodynamics applied to spacetime [9]. Besides, in the cosmological context such a conjecture allows to extract Friedmann equations by implementing the first law of thermodynamics on the apparent horizon of the Universe [10–13].

In the original formulation the gravity-thermodynamic conjecture applies Bekenstein-Hawking (BH) area law $S_{BH}=A/A_0$ to the Universe apparent horizon of surface area $A=4\pi r_{hor}^2$ and radius $r_{hor}$.^1^ Nevertheless, generalized forms of BH entropy have been discussed in recent literature, motivated by either nonextensive [14–16] or quantum gravity [17] arguments. To the latter class belongs Barrow entropy, which deforms BH area-law to

1 $$\begin{aligned} S\,=\,\left( \frac{A}{A_0}\right) ^{1+\Delta /2},\qquad 0\le \Delta \le 1, \end{aligned}$$

where Barrow exponent $\Delta$ embeds quantum gravitational corrections. In particular, $\Delta =1$ corresponds to the maximal departure from BH entropy, which is instead recovered for $\Delta =0$. Though being proposed for black holes [17], Eq. (1) is also applied within the cosmological framework, giving rise to modified Friedmann equations that predict a richer phenomenology comparing to the standard one [18]. In addition, one can rephrase the holographic principle in terms of Barrow entropy, obtaining Barrow holographic dark energy (BHDE) (see also [19–27] for recent applications of Barrow entropy in Cosmology). Comparison of the above constructions with observations sets upper limits on Barrow exponent [28–32], which slightly deviates from zero, as expected.

In physical cosmology, inflation is supposed to be a crucial era in the evolution of the Universe, consisting of a very short-lived, but extremely accelerated expansion phase occurred right after the Big Bang. Originally proposed in [33–36], it has been getting increasing attention over the years, becoming one of the two pillars of the present cosmological model along with the late time acceleration [37–39]. In spite of this, the origin of inflation has not been well understood yet. The most commonly adopted scenario is that it has been driven by a particular form of dark energy represented by a scalar field with slow rolling assumptions [40]. Alternative models have been recently proposed in [41–48]. The inflationary phase has also been studied in connection with holographic dark energy [49–52], motivated by the plausible role of the latter as a mechanism responsible for the late time cosmic acceleration.

Starting from the above premises, in this work we study the evolution and inflation of the Universe in the context of Barrow entropy-based Cosmology. In this sense, our analysis should be regarded as a preliminary attempt to explore the effects of quantum gravity on the dynamics of the Universe. In particular, we apply Barrow formula (1) to the entropy associated with the apparent horizon of a $(n+1)$-dimensional homogeneous and isotropic (Friedmann Robertson Walker-like) Universe, assuming that the matter inside the horizon is represented by a scalar field with a potential. In this setting, modified Friedmann equations are derived from the first law of thermodynamics and compared with the result of [22] for the specific case of $n=3$. Furthermore, we investigate the early inflationary dynamics of Barrow cosmology with the power-law potential function. Contrary to nonextensive (Tsallis-like) scenario [53], where it has been shown that inflation may consist of both slow roll- and kinetic-phases, here we find that only the first stage is eligible, the kinetic energy era being incompatible with the allowed values of Barrow exponent $\Delta$. After computing the characteristic inflation parameters, we infer an upper bound on $\Delta$ in compliance with recent observational constraints on the scalar spectral index and the tensor-to-scalar ratio. We finally comment on the consistency of our results with other approaches in literature aimed at exploring inflation driven by BHDE.

The remainder of the work is structured as follows: in the next Section, we derive modified Friedmann equations from Barrow entropy. Section 3 is devoted to to the study of the inflationary era in BHDE, while conclusions and outlook are summarized in Sect. 4.

## Modified Friedmann equations in barrow cosmology

Let us consider a homogenous and isotropic Friedmann–Robertson–Walker (FRW) Universe of spatial curvature *k*. We first set notation by following [22] and focusing on $(3+1)$-dimensions. To be as general as possible, the derivation of the modified Friedmann equations in Barrow Cosmology is then performed for the $(n+1)$-dimensional case, with $n\ge 3$.

For a $(3+1)$-dimensional FRW Universe, the line element can be written as

2 $$\begin{aligned} ds^2\,=\,h_{bc}dx^{b}dx^{c}+\tilde{r}^2\left( d\theta ^2+\sin ^2\theta \,d\phi ^2\right) , \end{aligned}$$

where we have denoted the metric of the $(1+1)$-dimensional subspace by $h_{bc}=\textrm{diag}[-1,a^2/(1-kr^2)]$. Moreover, $x^b=(t,r)$, $\tilde{r}=a(t)r$, *a*(*t*) is the (time-dependent) scale factor and *r* the comoving radius.

Following [54], the dynamical apparent horizon is obtained from the geometric condition

3 $$\begin{aligned} h^{bc}\partial _b\tilde{r} \,\partial _c \tilde{r}=0. \end{aligned}$$

For the FRW Universe (2), explicit calculations yield

4 $$\begin{aligned} \tilde{r}_A=\frac{1}{\sqrt{H^2+{k}/{a^2}}}, \end{aligned}$$

where $H=\dot{a}(t)/a(t)$ is the Hubble parameter and the overhead dot indicates derivative respect to the cosmic time *t*.

The apparent horizon has an associated temperature

5 $$\begin{aligned} T=\,\frac{\kappa }{2\pi }=-\frac{1}{2\pi \tilde{r}_A}\left( 1-\frac{\dot{\tilde{r}}_A}{2H\tilde{r}_A}\right) , \end{aligned}$$

where $\kappa$ represents the surface gravity. Clearly, for $\dot{\tilde{r}}_A\le 2H\tilde{r}_A$ we have $T\le 0$. To avoid meaningless negative temperatures, one can define $T = |\kappa |/2\pi$. Furthermore, it is possible to assume that $\dot{\tilde{r}}_A\ll 2H\tilde{r}_A$ in an infinitesimal time interval *dt*, which amounts to keeping the apparent horizon radius fixed. This implies the approximation $T\simeq 1/{2\pi \tilde{r}_A}$ [11].

We now suppose that the matter content of the Universe is represented by a scalar field $\phi$ characterized by a perfect fluid form. The corresponding Lagrangian is given by $\mathcal {L}_\phi =X-V(\phi )$, where $X=-\frac{1}{2}h^{\mu \nu }\partial _\mu \phi \partial _\nu \phi$ and $V(\phi )$ are the kinetic and (spatially homogenous) potential terms, respectively. In turn, the stress–energy tensor is

6 $$\begin{aligned} T_{\mu \nu }=(\rho _\phi +p_\phi )u_\mu u_{\nu }+p_\phi  h_{\mu \nu }, \end{aligned}$$

where $u_{\mu }$ is the four-velocity of the fluid and

7a $$\begin{aligned} \rho _\phi= & {} \frac{\dot{\phi }^2}{2}+V(\phi ), \end{aligned}$$

7b $$\begin{aligned} p_\phi= & {} \frac{\dot{\phi }^2}{2}-V(\phi ). \end{aligned}$$

represent its energy density and pressure, respectively [48]. In turn, the conservation equation $\nabla _{\mu }T^{\mu \nu }=0$ gives the continuity equation

8 $$\begin{aligned} \dot{\rho }_\phi +3H(\rho _\phi +p_\phi )=0. \end{aligned}$$

Combining Eqs. (7) and (8), we obtain the dynamics equation of the canonical scalar field as

9 $$\begin{aligned} \ddot{\phi }+3H\dot{\phi }+\partial _\phi V=0, \end{aligned}$$

where the term containing the Hubble constant serves as a kind of friction term resulting from the expansion.

### Modified Friedmann equations in $(n+1)$ dimensions

The above ingredients provide the basics to derive the modified Friedmann equations in Barrow entropy-based Cosmology. Following [55], here we extract such equations from the first law of thermodynamics

10 $$\begin{aligned} dE\,=\,TdS+W dV, \end{aligned}$$

applied to the apparent horizon of the FRW Universe in $(n+1)$-dimensions, where

11 $$\begin{aligned} W=(\rho _\phi -p_\phi )/2, \end{aligned}$$

is the work density associated to the Universe expansion and

12 $$\begin{aligned} S\,=\,\gamma \left( \frac{A}{A_0^{(n-1)/2}}\right) ^{1+\Delta /2}, \end{aligned}$$

is the generalized Barrow entropy. We have denoted the *n*-dimensional horizon surface by $A=n\Omega _n\tilde{r}_A^{n-1}$, where $\Omega _n\,\equiv \,\frac{\pi ^{n/2}}{\Gamma (n/2+1)}$ is the angular part of the *n*-dimensional sphere and $\Gamma$ the Euler’s function. The dimensionless constant $\gamma$ is such that $\gamma \rightarrow 1$ for $n=3$, so that Eq. (1) is restored in this limit. Its explicit expression shall be fixed later. In passing, we mention that an alternative derivation of modified Friedmann equations can be built upon Padmanabhan’s paradigm of emergent gravity [56], which states that the spatial expansion of our Universe can be understood as the consequence of the emergence of space with the progress of cosmic time.

Now, by taking into account that the total energy of the Universe inside the *n*-dimensional volume $V=\Omega _n\tilde{r}_A^n$ is $E=\rho _\phi V$, we have

13 $$\begin{aligned} dE= Vd\rho _\phi + \rho _\phi dV=\Omega _n \tilde{r}_A^n \dot{\rho }_\phi dt \,+\, \rho _\phi  \Omega _n n\tilde{r}_A^{n-1}d\tilde{r}_A. \end{aligned}$$

This relation can be further manipulated by resorting to the generalized continuity equation

14 $$\begin{aligned} \dot{\rho }_\phi +nH(\rho _\phi +p_\phi )=0, \end{aligned}$$

to give

15 $$\begin{aligned} dE\,=\,-\Omega _n \tilde{r}_A^n n H \left( \rho _\phi +p_\phi \right) dt \,+\, \rho _\phi  \Omega _n n\tilde{r}_A^{n-1}d\tilde{r}_A. \end{aligned}$$

On the other hand, by differentiating the entropy (12) we get

16 $$\begin{aligned} dS= & {} \gamma \left( \frac{1}{A_0^{(n-1)/2}}\right) ^{1+\Delta /2}n\Omega _n\left( 1+\frac{\Delta }{2}\right) \left( n-1\right) \nonumber \\{} & {} \quad \times \left( n\Omega _n\tilde{r}_A^{n-1}\right) ^{\Delta /2}\tilde{r}_A^{n-2}d\tilde{r}_A. \end{aligned}$$

By plugging Eqs. (13)–(16) into (10), we arrive to

17 $$\begin{aligned} H\left( \rho _\phi +p_\phi \right) dt= & {} \frac{\gamma \left( n-1\right) \left( 1+\frac{\Delta }{2}\right) \left( n\Omega \tilde{r}_A^{n-1}\right) ^{\Delta /2}}{2\pi \tilde{r}_A^3}\nonumber \\{} & {} \quad \times \left( \frac{1}{A_0^{(n-1)/2}}\right) ^{1+\Delta /2}\,dr. \end{aligned}$$

With the further use of the continuity Eq. (14), this becomes

18 $$\begin{aligned} -\frac{2\pi \left( A_0^{(n-1)/2}\right) ^{1+\Delta /2}}{\gamma \,n\left( n-1\right) (1+\frac{\Delta }{2})\left( n\Omega \right) ^{\Delta /2}} \,d\rho _\phi \,=\,\tilde{r}_A^{(n-1)\Delta /2-3}d\tilde{r}_A. \end{aligned}$$

Integrating both sides, we are led to

19 $$\begin{aligned}{} & {} \tilde{r}_A^{(n-1)(1+\Delta /2)-n-1}\nonumber \\{} & {} =\frac{\pi \left[ 4-\left( n-1\right) \Delta \right] \left( A_0^{(n-1)/2}\right) ^{1+\Delta /2}}{\gamma n\left( n-1\right) \left( 1+\frac{\Delta }{2}\right) \left( n\Omega \right) ^{\Delta /2}}\,\rho _\phi , \end{aligned}$$

where the integration constant has been fixed by imposing the boundary condition $8\pi \rho _{\phi }=\Lambda \simeq 0$. Finally, with the help of the definition (4), we obtain

20 $$\begin{aligned} \left( H^2+\frac{k}{a^2}\right) ^{1-(n-1)\Delta /4}\,=\,\frac{8\pi G_{eff}^{(n-1)/2}}{3}\sigma \rho _\phi , \end{aligned}$$

where we have defined

21 $$\begin{aligned} \sigma \equiv \frac{3}{n-2}\,\frac{\left[ n+1-\left( n-1\right) \left( 1+\frac{\Delta }{2}\right) \right] }{n\left( 2-\Delta \right) }, \end{aligned}$$

and we have set

22 $$\begin{aligned} \gamma \,{=}\,\frac{\pi ^{(n-1)\Delta /4}}{2\left( n\Omega _n\right) ^{\Delta /2}\,4^{(1+\Delta /2)(1-n)/2}}\,\frac{\left( n-2\right) }{\left( n{-}1\right) }\left( \frac{2{-}\Delta }{2{+}\Delta }\right) ^{(3{-}n)/2}. \end{aligned}$$

Furthermore, we have introduced the effective gravitational constant [22]

23 $$\begin{aligned} G_{eff}\,=\,\frac{A_0}{4}\left( \frac{2-\Delta }{2+\Delta }\right) \left( \frac{A_0}{4\pi }\right) ^{\Delta /2}. \end{aligned}$$

Some comments are in order here: first, we notice that for $n=3$, we have $\gamma \rightarrow 1$, consistently with the discussion below Eq. (12). The same is true for $\sigma$, so that Eq. (20) for $n=3$ becomes

24 $$\begin{aligned} \left( H^2+\frac{k}{a^2}\right) ^{1-\Delta /2}\,=\,\frac{8\pi G_{eff}}{3}\rho _\phi . \end{aligned}$$

This is nothing but the first modified Friedmann equation derived in [22] when $\rho _\phi \equiv \rho$ (normal matter). Furthermore, the limit $\Delta \rightarrow 0$ correctly reproduces the standard Friedmann equation

25 $$\begin{aligned} H^2+\frac{k}{a^2}\,=\,\frac{8\pi }{3}\frac{A_0}{4}\rho _\phi . \end{aligned}$$

As a final remark, it must be emphasized that, due to the positive definition of the energy density, Eqs. (20) and (21) imply the upper bound

26 $$\begin{aligned} n+1-\left( n-1\right) \left( 1+\frac{\Delta }{2}\right) >0 \,\,\,\, \Longrightarrow \,\,\,\, \Delta <\frac{4}{n-1}, \end{aligned}$$

which is obviously satisfied for any allowed value of *n*.

Now, from the time derivative of Eq. (24), one can easily obtain the second modified Friedmann equation as follows

27 $$\begin{aligned}{} & {} 2H\left[ 1-\left( n-1\right) \frac{\Delta }{4}\right] \left( H^2+\frac{k}{a^2}\right) ^{-(n-1)\Delta /4}\nonumber \\{} & {} \quad \times \left( \frac{\ddot{a}}{a}-H^2-\frac{k}{a^2}\right) =\frac{8\pi G_{eff}^{(n-1)/2}}{3}\sigma \dot{\rho }_\phi . \end{aligned}$$

By use of the continuity Eq. (14), this gives

28 $$\begin{aligned}{} & {} \left[ 1-\left( n-1\right) \frac{\Delta }{4}\right] \left( H^2+\frac{k}{a^2}\right) ^{-(n-1)\Delta /4}\nonumber \\{} & {} \quad \times \left( \frac{\ddot{a}}{a}-H^2-\frac{k}{a^2}\right) =-\frac{4\pi G_{eff}^{(n-1)/2}}{3}\,\sigma n \left( \rho _\phi +p_\phi \right) .\nonumber \\ \end{aligned}$$

Replacing $\rho _\phi$ by the first Friedmann equation (20), we find after some simplification

29 $$\begin{aligned}{} & {} \left[ 4-\left( n-1\right) \Delta \right] \frac{\ddot{a}}{a}\left( H^2+\frac{k}{a^2}\right) ^{-(n-1)\Delta /4}\nonumber \\{} & {} \quad +\left[ 2n-4+\Delta \left( n-1\right) \right] \left( H^2+\frac{k}{a^2}\right) ^{1-(n-1)\Delta /4}\nonumber \\{} & {} =-\frac{16\pi G_{eff}^{(n-1)/2}}{3}\,\sigma  n p_\phi . \end{aligned}$$

This is the second modified Friedmann equation in Barrow Cosmology. Again, one can check that $n=3$ gives back the result of [22]

30 $$\begin{aligned}{} & {} \left( 2-\Delta \right) \frac{\ddot{a}}{a}\left( H^2{+}\frac{k}{a^2}\right) ^{{-}\Delta /2}{+} \left( 1{+}\Delta \right) \left( H^2{+}\frac{k}{a^2}\right) ^{1{-}\Delta /2}\,\nonumber \\{} & {} \quad =\,-8\pi G_{eff}\,p_\phi . \end{aligned}$$

The further limit $\Delta \rightarrow 0$ reproduces the standard second Friedmann equation, here rewritten as

31 $$\begin{aligned} \dot{H}+H^2=-\frac{4\pi }{3}\left( \rho _\phi +3p_\phi \right) , \end{aligned}$$

where we have used the relation

32 $$\begin{aligned} \dot{H}=\frac{\ddot{a}}{a}-H^2. \end{aligned}$$

## Inflation in barrow cosmology

Let us now move onto the study of the inflationary era of the Universe. Within the scalar theory framework considered above, the characteristic quantities to compute are the inflation slow-roll parameters, which are defined by

33 $$\begin{aligned} \epsilon= & {} - \frac{\dot{H}}{H^2}, \end{aligned}$$

34 $$\begin{aligned} \eta= & {} -\frac{\ddot{H}}{2H\dot{H}}. \end{aligned}$$

Slow-roll conditions assert that both these two parameters take very small values during inflation, i.e. $\epsilon ,\eta \ll 1$. In the slow-roll theoretical framework, the only requirement $\epsilon \ll 1$ is actually needed to ensure the existence of an early inflationary era, Then, by imposing $\dot{\phi }^2,\ddot{\phi }\ll 1$ on the equation of motion of the theory, the first Friedmann equation (20) under the slow-roll assumptions becomes

35 $$\begin{aligned} H^2\simeq \left[ \frac{8\pi G_{eff}}{3}\, V(\phi )\right] ^{2/(2-\Delta )}, \end{aligned}$$

where we have focused on the case $n=3$ and we have resorted to Eq. (7a).

On the other hand, from the second Friedmann equation (30) we get

36 $$\begin{aligned} \dot{H}\simeq \frac{3\dot{\phi }^2}{2\left( \Delta -2\right) }\left( \frac{8\pi G_{eff}}{3}\right) ^{2/(2-\Delta )}V(\phi )^{\Delta /(2-\Delta )}. \end{aligned}$$

Combining Eqs. (35) and (36), the slow-roll parameters (33) and (34) take the form

37 $$\begin{aligned} \epsilon\simeq & {} \frac{3\dot{\phi }^2}{2\left( 2-\Delta \right) }V(\phi )^{-1}, \end{aligned}$$

38 $$\begin{aligned} \eta\simeq & {} -\left( \frac{8\pi G_{eff}}{3}V(\phi )\right) ^{1/(\Delta -2)} \left[ \frac{\ddot{\phi }}{\dot{\phi }}+\frac{\dot{\phi }\,\Delta }{4-2\Delta }\frac{\partial _\phi V(\phi )}{V(\phi )}\right] . \nonumber \\ \end{aligned}$$

Let us now remark that the above parameters should be computed at horizon crossing, where the fluctuations of the inflation field freeze [53].

The scalar spectral index of the primordial curvature perturbations and the tensor-to-scalar ratio are defined by

39 $$\begin{aligned} n_s\simeq & {} 1-6\epsilon +2\eta , \end{aligned}$$

40 $$\begin{aligned} r\simeq & {} 16\epsilon , \end{aligned}$$

respectively, which also need to be evaluated at the horizon crossing. For later convenience, it is useful to introduce the e-folding time

41 $$\begin{aligned} N=\int _{t_i}^{t_f} H(t) dt, \end{aligned}$$

where $t_i$ $(t_f)$ represents the initial (final) time of the inflationary era. Consistently with the above discussion, we consider $t_i=t_c$ as the horizon crossing time, so that Eq. (41) can be rewritten as $N=\int _{\phi _c}^{\phi _f} H{\dot{\phi }}^{-1} \,d\phi$, where we have used the notation $\phi _c\equiv \phi (t_c)$ and $\phi _f\equiv \phi (t_f)$.

### Slow-roll inflation with power-law potential

We now examine inflation from the dynamical point of view. Toward this end, we assume a power-law behavior for the scalar potential $V(\phi )$ in the form

42 $$\begin{aligned} V(\phi )\simeq \phi ^{m}, \end{aligned}$$

where $m>0$ is the power-term. The latest observational data prefer models with $m\sim \mathcal {O}(10^{-1})$ or $m\sim \mathcal {O}(1)$, although $m\ge 2$ is disfavored in the minimally coupled scalar field.^2^ In what follows, we focus on the $m\sim \mathcal {O}(1)$ scenario. We also remark that power law inflation is a very useful model to assess approximation schemes for the computation of scalar power spectra, since its spectrum is exactly solvable.

In order to extract analytical solutions of the inflationary observable indices, we express $\dot{\phi }$ and $\ddot{\phi }$ in terms of the scalar field by using the slow-roll conditions. In this regard, let us observe that the evolution Eq. (9) can be rewritten as

43 $$\begin{aligned} \dot{\phi }\simeq -\frac{1}{3H}\partial _\phi V. \end{aligned}$$

By plugging into (35), we get

44 $$\begin{aligned} \dot{\phi }=-\frac{m}{3}\left( \frac{8\pi G_{eff}}{3}\right) ^{1/(\Delta -2)}\,\phi ^{\left[ \left( 2-\Delta \right) \left( m-1\right) -m\right] /\left( 2-\Delta \right) }. \end{aligned}$$

We can now derive the expression of $\phi _f$ by noticing that inflation is supposed to end when $\epsilon (\phi _f)\sim 1$. By inverting Eq. (37), we are led to

45 $$\begin{aligned} \phi _f=\left[ \frac{6\left( 2-\Delta \right) }{m^2}\left( \frac{8\pi G_{eff}}{3}\right) ^{2/(2-\Delta )} \right] ^{(2-\Delta )/[\Delta (2-m)-4]}. \end{aligned}$$

Similarly, insertion of Eqs. (35) and (44) into (41) allows to infer the following expression for the scalar field at horizon crossing

46 $$\begin{aligned} \phi _c= & {} \Bigg \{ \frac{m}{3\left( 2-\Delta \right) }\left( \frac{8\pi G_{eff}}{3}\right) ^{2/(\Delta -2)}\nonumber \\{} & {} \Bigg \{\frac{m}{2}+N\left[ 4+\Delta \left( m-2\right) \right] \Bigg \}\Bigg \}^{(2-\Delta )/\left[ 4+\Delta (m-2)\right] }. \end{aligned}$$

The scalar spectral index (39) and the tensor-to-scalar ratio (40) can be cast in terms of the power-term *m* and the e-folding time *N* as

47 $$\begin{aligned} n_s\simeq & {} 1-\frac{2\left[ 4+\Delta (m-2)+m\right] }{m\left\{ 1+\frac{2N}{m}\left[ 4+\Delta (m-2)\right] \right\} }, \end{aligned}$$

48 $$\begin{aligned} r\simeq & {} \frac{16}{1+\frac{2N}{m}\left[ 4+\Delta (m-2)\right] }. \end{aligned}$$

Remarkably, we see that the slow-roll indices only depend on the power-term *m* and Barrow parameter $\Delta$. A similar result has been exhibited in the context of Tsallis deformation of entropy-area law [53].

In order to constrain Barrow exponent $\Delta$, let us require consistency of Eqs. (47) and (48) with observations. Specifically, we consider Planck 2018 measurements, which set the following bounds on $n_s$ and *r* [57]

49 $$\begin{aligned} n_s= & {} 0.9649 \pm 0.0042 \,\,\,\, (68\%\,\, \textrm{CL}) \,\,\,\, \nonumber \\{} & {} \mathrm {from\,\,Planck\,\, TT,TE,EE+lowE+lensing}, \end{aligned}$$

50 $$\begin{aligned} r< & {} 0.064  (95\%\,\, \textrm{CL}) \,\,\,\, \nonumber \\{} & {} \mathrm {from\,\, Planck\,\, TT,TE,EE+lensing+lowEB}. \end{aligned}$$

**Fig. 1:**
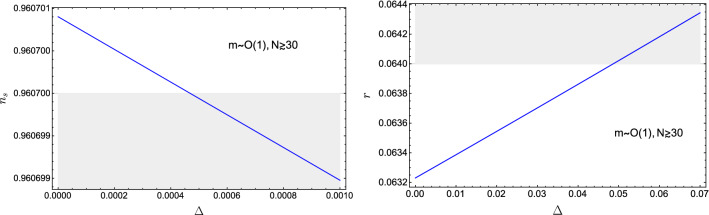
Plot of $n_s$ (left panel) and *r* (right panel) versus the Barrow parameter $\Delta$. The shaded region is excluded by the observational constraints (49)-(50)

Figure 1 shows the plot of $n_s$ (left panel) and *r* (right panel) as a function of $\Delta$. For sufficiently long inflation ($N\gtrsim 30$ e-folds) and $m\sim \mathcal {O}(1)$, we see that, while $\Delta \lesssim \mathcal {O}(10^{-2})$ would be still allowed by the experimental bound (50) on *r*, the constraint (49) on $n_s$ necessarily requires $\Delta \lesssim \mathcal {O}(10^{-4})$. We would like to emphasize that the best we can do at this stage is to provide the order of magnitude of the bound on $\Delta$, rather than its exact value, since we are only considering an estimate of the order of magnitude of other model parameters, such as the power-term *m*. Thus, deviations from zero of Barrow parameter higher than the above threshold appear phenomenologically incompatible with the description of the slow-roll inflation with power-law potential. Clearly, the above result could be improved allowing *N* and *m* to vary too and applying suitable simulation techniques. We reserve this technical analysis for future study.

For comparison with recent literature on Barrow entropy, we notice that the obtained bound $\Delta \lesssim \mathcal {O}(10^{-4})$ improves the constraints $\Delta =0.0094^{+0.094}_{-0.101}$ and $\Delta \lesssim 0.08$ derived through Supernovae (SNIa) Pantheon sample [28, 29] and baryogenesis [32] measurements, respectively, and is consistent with the most stringent bound $\Delta \lesssim 1.4\times 10^{-4}$ found through Big Bang Nucleosynthesis [30].

### Kinetic inflation with power-law potential

Above we have argued that the slow-roll inflation terminates when $\epsilon \sim 1$. Two scenarios can then occur: either the scalar field oscillates to the minimal value of the potential, leading the Universe into a decelerated expansion phase,or the inflation goes on but with different features. Here, we shall examine whether the latter possibility is allowed within Barrow entropy-based Cosmology. In particular, a crucial assumption of the slow-roll inflation is that the kinetic energy of the scalar field can be neglected. However, if the volume of the Universe is large enough before the field starts to oscillate, then a kinetic term might arise and drive a transition from a vacuum state to quintessence. We assume the kinetic contribute in the form

51 $$\begin{aligned} \dot{\phi }^2=m V(\phi ). \end{aligned}$$

The above expression can be actually deduced from the dynamics relation (9) and the modified Friedmann equations (24) and (30), here rewritten for convenience as

52 $$\begin{aligned} H^2= & {} \left( \frac{8\pi G_{eff}}{3}\right) ^{2/(2-\Delta )}\left[ \frac{\dot{\phi }^2}{2}+V(\phi )\right] ^{2/(2-\Delta )}, \end{aligned}$$

53 $$\begin{aligned} \dot{H}= & {} \frac{3}{\Delta -2}\dot{\phi }^2\left( \frac{8\pi G_{eff}}{3}\right) ^{2/(2-\Delta )}\left( \frac{\dot{\phi }^2}{2}+V\right) ^{\Delta /(2-\Delta )}. \end{aligned}$$

These equations also allow us to express the slow-roll parameters as

54 $$\begin{aligned} \epsilon= & {} \frac{6m}{\left( 2-\Delta \right) \left( m+2\right) }, \end{aligned}$$

55 $$\begin{aligned} \eta= & {} \frac{m^{3/2}}{(\Delta -2)}\, \left( \frac{8\pi G_{eff}}{3}\right) ^{1/(\Delta -2)} \left( \frac{m+2}{2}\right) ^{1/(\Delta -2)}\nonumber \\{} & {} \times \phi ^{\left[ \Delta (2-m)-4\right] /\left[ 2(2-\Delta )\right] }. \end{aligned}$$

Now, the end of the kinetic inflation is set by the condition $\eta (\phi _f)\simeq 1$ [53], which gives from the definition (41)

56 $$\begin{aligned} \phi _c= & {} \Bigg \{ \left( \frac{8\pi G_{eff}}{3}\right) ^{1/(\Delta -2)} \left( \frac{m+2}{2}\right) ^{1/(\Delta -2)}\frac{m^{1/2}}{2(\Delta -2)}\nonumber \\{} & {} \quad \times \left[ 2m+ N\left[ 4+\Delta \left( m-2\right) \right] \right] \Bigg \} ^{2(2-\Delta )/[4+\Delta (m-2)]}.\nonumber \\ \end{aligned}$$

From Eqs. (54) and (55), we then get

57 $$\begin{aligned}{} & {} n_s{=}\left\{ 1 {+} 4m\, \left\{ \frac{9}{\left( m{+}2\right) \left( \Delta {-}2\right) }{+}\frac{\left\{ \frac{m^{1/2}}{2(\Delta {-}2)}\left( \frac{m{+}2}{2}\right) ^{1/(\Delta {-}2)}\left( \frac{8\pi G_{eff}}{3}\right) ^{1/(\Delta {-}2)}\left[ 2m{+}N\left[ 4{+}\Delta \left( m{-}2\right) \right] \right] \right\} ^{n\Delta /[2(\Delta {-}2)]}}{2m{+}N\left[ 4{+}\Delta \left( m{-}2\right) \right] } \right\} \right\} ^{2(2{-}\Delta )/[4{+}\Delta (m{-}2)]}, \nonumber \\ \end{aligned}$$

58 $$\begin{aligned} r=\frac{96m}{\left( m+2\right) \left( 2-\Delta \right) }. \end{aligned}$$

**Fig. 2:**
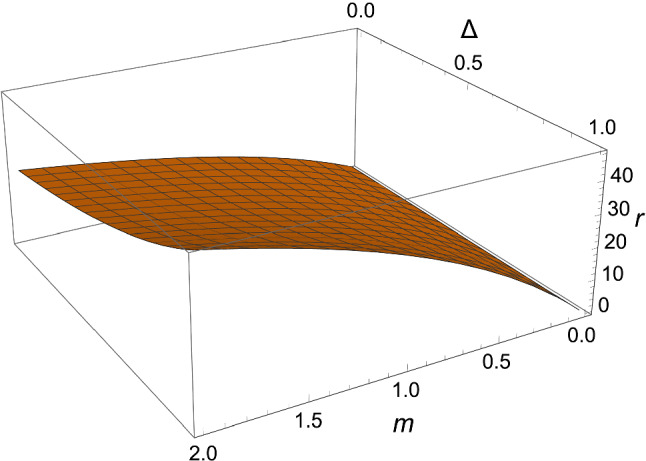
3D plot of *r* versus the power-term *m* and Barrow parameter $\Delta$

Unlike the previous scenario, we now find that observational consistency for $m\sim \mathcal {O}(1)$ is obtained, provided that $\Delta$ assumes largely negative values. This can be easily seen from Eq. (58) (see also the plot in Fig. 2). The same occurs for $m\sim \mathcal {O}(10^{-1})$. However, such a condition is at odds with the assumption (1), apparently implying that a kinetic-like inflation may not be explained within Barrow’s framework. This is a remarkable difference with the case of inflation based on Tsallis entropy [53], which allows for kinetic phase too. Specifically, in that case the kinetic inflation is associated to a regime of decreasing horizon entropy and ensuing clumping of fluctuations in particular regions of spacetime.

## Discussion and conclusions

Inspired by Covid-19 fractal structure, the modified entropy-area law (1) has been proposed by Barrow to take into account quantum gravitational effects on the black hole horizon surface [17]. In the lines of the gravity-thermodynamic conjecture, this paradigm has been applied to the Universe horizon too, the ensuing framework being known as Barrow Cosmology. Within this framework, we have studied the evolution of FRW Universe, assuming the matter content to be represented by a homogeneous scalar field in the form of a perfect fluid. As a first step, by using the first law of thermodynamics applied to the horizon of the FRW Universe, we have derived modified ($\Delta$-dependent) Friedmann equations. The obtained result has been used to analyze the inflationary era. Toward this end, we have supposed a power-law behavior for the scalar inflaton field. We have found that inflation in Barrow Cosmology can consist of the slow-roll phase only, the kinetic inflation being incompatible with the allowed values of Barrow deformation parameter. We have finally constrained Barrow exponent to $\Delta \lesssim \mathcal {O}(10^{-4})$ by demanding consistency of the scalar spectral index and tensor-to-scalar ratio with recent observational Planck data.

Other aspects deserve further analysis. Besides the background and inflationary evolution, it would be interesting to study the growth rate of matter density perturbations and structure formation. This is an important testing ground to discriminate among existing modified cosmological models. Preliminary investigation in this direction has been proposed in [58] in the context of both Tsallis and Barrow entropies, showing that the entropic deformation parameter significantly influences the growth of perturbations. Moreover, one can attempt to extend the present considerations to Cosmology based on other deformed entropies, such as Tsallis entropy with or without radiation sector [59], Kaniadakis entropy [60], which is a self-consistent relativistic generalization of Boltzmann–Gibbs entropy with non-trivial cosmological implications [61], Rényi [62] or Sharma-Mittal [63] entropy, which both arise in the context of information theory. Finally, since our models is an effort to include quantum gravity corrections in the analysis of inflation, it is essential to examine the obtained results in connection with predictions from more fundamental theories of quantum gravity [64]. Work along these and other directions is under active consideration and will be presented elsewhere.

## Data Availability

This manuscript has no associated data or the data will not be deposited. [Authors’ comment: Data sharing is not applicable to this article as no datasets were generated or analyzed during the current study.]

## Footnotes

1 Here and henceforth we work in natural units $\hslash =c=G=k_B=1$. Accordingly, the Planck area is $A_0=4$.

2 More generally, one can assume $V(\phi )=V_0\phi ^{m}$, where $V_0$ is a positive constant with dimensions of $[E]^{4-m}$. However, since observational indices are shown to be independent of this quantity, we can set $V_0=1$ in suitable units without loss of generality.
